# Improved Lithium Storage Performance of a TiO_2_ Anode Material Doped by Co

**DOI:** 10.3390/ma16041325

**Published:** 2023-02-04

**Authors:** Li Cai, Fang-Chao Gu, Shu-Min Meng, An-Qi Zhuang, Hang Dong, Zi-Zhe Li, Zhen-Feng Guan, De-Shuai Li, Yong Li, Xi-Xiang Xu, Qiang Li, Qiang Cao

**Affiliations:** 1 Spintronics Institute, University of Jinan, Jinan 250022, China; 2 College of Physics, Weihai Innovation Research Institute, Institute of Materials for Energy and Environment, Qingdao University, Qingdao 266071, China

**Keywords:** TiO_2_, Co ion doping, lithium-ion batteries, lithium storage performance, kinetic analysis

## Abstract

TiO_2_ is a promising anode material for lithium-ion batteries (LIBs) due to its low cost, suitable operating voltage, and excellent structural stability. The inherent poor electron conductivity and low ion diffusion coefficient, however, severely limit its application in lithium storage. Here, Co-doped TiO_2_ is synthesized by a hydrothermal method as an anode material since Co@TiO_2_ possesses a large specific surface area and high electronic conductivity. Thanks to the Co dopants, the ion diffusion and electron transport are both greatly improved, which is very beneficial for cycle stability, coulombic efficiency (CE), reversible capacity, and rate performance. As a result, Co@TiO_2_ shows a high reversible capacity of 227 mAh g^−1^ at 3 C, excellent rate performance, and cycling stability with a capacity of about 125 mAh g^−1^ at 10C after 600 cycles (1 C = 170 mA g^−1^).

## 1. Introduction

Lithium-ion batteries have been widely used in various electronic devices, owing to their high energy density, excellent cycle stability, and environmental friendliness [1,2,3,4,5]. In recent years, they have been considered one of the most promising power sources for electrical vehicles and new energy storage in order to reduce costs and consider the impact on the environment. Graphite, as an important anode electrode material, has been used in commercial LIBs. However, it easily forms lithium dendrites at a low operating voltage, causing a series of safety problems [6]. Moreover, it undergoes an irreversible capacity loss during initial discharge due to the formation of a solid electrolyte interphase (SEI) film [7,8]. Therefore, LIBs urgently need new anode materials with reinforced electrochemical performance. It is worth noting that TiO_2_ is a promising anode material candidate because of its excellent operational safety, good reversible capacity, environmental benignity, suitable operating voltage, and superior structural stability [5,9]. However, it has inherent poor electronic conductivity and low ion diffusion rate, which seriously limit its actual electrochemical performance. In particular, the low theoretical capacity seriously affects its practical application in business [10].

To enhance the lithium storage performance of LIBs, several effective strategies have been proposed [11,12]. The most common way is to adjust the shape on the nanometer scale, such as nanowires/nanofibers [13,14], nanorods [15,16], nanospheres [17,18,19], and nanotubes [20,21,22]. These special morphologies with large specific surface areas can provide more active surface sites for electrolyte permeation and lithium storage. Second, to improve the diffusion rate of lithium ions, a practical method is to synthesize nanoscale materials, which greatly reduces the diffusion distance, thereby effectively shortening the diffusion time of lithium ions [23]. Then, the composite materials can be formed by combining with other transition metal oxides, such as iron oxide [24] and nickel oxide [25,26], due to their higher theoretical capacity. In addition, some methods are proposed to further enhance the conductivity of TiO_2_. For example, the combination of carbon nanotubes, graphite, and other conductive additives is used to form some composite materials [27,28,29,30,31,32,33]. Although these conductive additives can effectively improve the electron transport rate on the surface of the particles, they still suffer from low conductivity in TiO_2_.

Here, a Co-doped anatase TiO_2_ composite with excellent lithium storage capacity was synthesized by an economical wet chemistry process. Co atoms replace some Ti atoms, which can not only effectively inhibit the growth of TiO_2_ crystals, but also reduce the band gap, thereby enhancing the electronic conductivity. Moreover, the charge-transfer resistance was decreased. In the meanwhile, the lithium diffusion coefficient was increased by Co doping. Therefore, the Co@TiO_2_ exhibits excellent electrochemical performance, which is promising for the anode material of LIBs.

## 2. Experimental

### 2.1. Materials and Synthesis

Co-doped TiO_2_ was synthesized via a hydrothermal method, which includes hydrolysis of tetrabutyl titanate (TBT) in a mixture of ethylene glycol and CoCl_2_·6H_2_O, followed by heating at 500 °C. Typically, 0.42 g CoCl_2_·6H_2_O was homogeneously dispersed in 50 mL ethylene glycol stirred for 2 h, and then 5 mL TBT was added to a uniformly distributed yellow solution and stirred vigorously for 2 h. After that, a Teflon-lined autoclave was used to seal the mixture and kept for 5 h at 180 °C. Then, the yellow precipitate was collected and dried in a vacuum oven at 80 °C for 12 h. Finally, the sample was annealed at 500 °C for 3 h under an air atmosphere. This product is marked as Co@TiO_2_. By comparison, undoped TiO_2_ was prepared under the same conditions except with CoCl_2_·6H_2_O as an additive.

### 2.2. Materials Characterization

The morphology of the materials was characterized by scanning electron microscopy (SEM, JSM-6700F) (JEOL, Beijing, China). The morphology and microstructure were further investigated by transmission electron microscopy (TEM) and HRTEM (JEOL JEM-2100F) (JEOL, Beijing, China). Energy-dispersive X-ray spectroscopy (EDS) was assembled with SEM. Nitrogen adsorption/desorption isotherms were registered on an ASAP 2020 instrument (Micrometrics, Norcross, GA, USA). The specific surface area and pore size distribution were calculated using the Brunauer–Emmett–Teller (BET) and Barrett–Joyner–Halenda (BJH) theories. The crystal structures were performed by X-ray diffraction (XRD) (Bruker, Billerica, MA, USA) with Cu Kα radiation. The valence state of Co@TiO_2_ was determined by X-ray photoelectron spectroscopy (XPS) using a Thermo Scientific ESCALAB 250XI photoelectron spectrometer with a wavelength of 450 nm (Thermo Fisher Scientific, Wuhan, China). The magnetic properties were investigated by a Quantum Design PPMS magnetometer at ambient temperature. The applied field dependence of magnetization was measured with steps of 0.01 and 0.01 T in the ranges from −0.05 to 0.05 T and from ±0.05 to ±3 T, respectively.

### 2.3. Electrochemical Measurements

The electrochemical properties of the samples were measured by CR-2032 coin-type cells. The working electrode, coated on the copper foil and then dried at 70°C for 12 h in a vacuum oven, was composed of active material (TiO_2_ or Co@TiO_2_), conductive carbon black (Super P), and sodium carboxymethyl cellulose (CMC) at a weight ratio of 7:2:1. The electrodes were pressed into disks (10 mm, diameter). The average active mass loading of the electrode is 1.5–2.0 mg cm^−1^ on current collectors. The coins were assembled in an argon-filled glove box (<0.1 ppm of H_2_O, <0.1 ppm of O_2_). The electrolyte was 1 M LiPF_6_ dissolved in a 1:1:1 mixture of ethylene carbonate (EC)/dimethyl carbonate (DMC)/diethyl carbonate (DEC). Celgard 2032 film was utilized as the separator (Whatman). Lithium metal was used as the reference electrode in LIBs. The discharge/charge measurements were performed on a NEWARE battery testing system at various current rates between 0.01 and 3.0 V vs. Li/Li^+^. Cyclic voltammetry (CV) measurement was performed on a CHI660E electrochemical workstation (CH Instruments Inc., Shanghai, China) at a scan rate of 0.5 mV·s^−1^ between 0.01 and 3.0 V. Electrochemical impedance spectroscopy (EIS) was conducted at open circuit potential state in the frequency range of 0.01–100 kHz with 5 mV amplitude on a CHI660E electrochemical workstation (CH Instruments Inc., Shanghai, China). The galvanostatic intermittent titration technique (GITT) method proposed by Weppner et al., as a reliable technique with highly resolved data, was used to determine the Li^+^ ions diffusion coefficient (*D*_Li_) in the electrode materials [34]. The *D*_Li_ can be calculated by the following equation [35,36]:

$$D_{Li^{+}}=\frac{4}{\pi }{\left(\frac{mV_{M}}{MS}\right)}^{2}{\left(\frac{\Delta E_{s}}{\Delta E_{\tau }}\right)}^{2}$$

where the *m* (g) is mass, the *V_M_* (cm^3^ mol^−1^) and *M* (g mol^−1^) are the molar volume of the active material and molecular weight, respectively. S is the surface area of the electrode. 

## 3. Results and Discussion

### 3.1. Materials Characterizations

**Figure 1:**
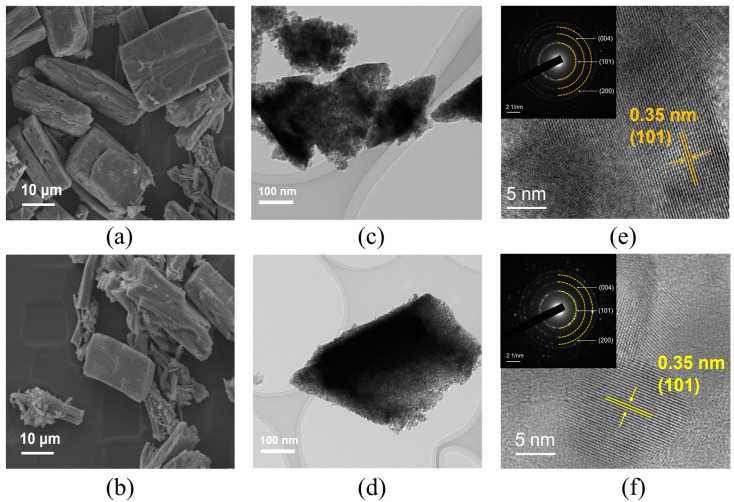
(**a**) SEM, (**c**) TEM, and (**e**) HRTEM images of TiO_2_. (**b**) SEM, (**d**) TEM, and (**f**) HRTEM images of Co@TiO_2_. The insets in (**e**,**f**) are the TiO_2_- and Co@TiO_2_-related SAED patterns, respectively.

The morphology and structure of the pristine TiO_2_ and Co@TiO_2_ materials were confirmed by SEM, TEM, and HRTEM images, as shown in Figure 1. Figure 1a,b display the SEM images of TiO_2_ and Co@TiO_2_, respectively. Obviously, both before and after doping the materials possess similar morphology that looks like an irregular prism of high quality. TEM (Figure 1c) shows that TiO_2_ is composed of a large number of tiny nanocrystals (8–12 nm). HRTEM (Figure 1e) shows a lattice fringe of 0.35 nm, which can be marked as the (101) crystal plane spacing of the anatase phase. Furthermore, the inset of Figure 1e exhibits the SAED pattern, which demonstrates the desired phase of the sample. The insert ring pattern in Figure 1f formed by small diffraction spots indicates a nanocrystalline structure of the irregular prism. In fact, the irregular prism image (Figure 1d) was made up of tiny nanocrystal subunits with a diameter of 7–10 nm. The crystallite sizes for all the samples were calculated using Scherrer’s formula [37]. In the comparative experiment, it is worth noting that the modified product shows a smaller size. This may be attributed to the dopants’ inhibition of the growth of the TiO_2_ crystal [38].

**Figure 2:**
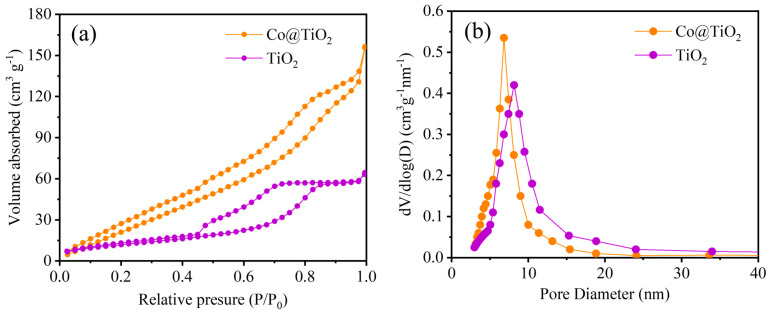
(**a**) N_2_ adsorption–desorption curves and (**b**) pore size distribution of TiO_2_ and Co@TiO_2_.

In order to prove the specific surface area and pore size distribution of the products, the N_2_ adsorption–desorption isotherm measurements were carried out, as shown in Figure 2. Note that both of them exhibit a typical type-IV isotherm. Figure 2a,b show the specific surface area and pore size distribution, respectively. Obviously, both represent mesoporous properties. The specific surface area of Co@TiO_2_ is 131.929 m^2^ g^−1^, which is larger than that of the TiO_2_ sample at 92.251 m^2^ g^−1^. Thanks to the dopants, the growth of the TiO_2_ crystal will be inhibited to form smaller particles, thus having a larger specific surface area [27,39]. From the pore size distribution, it can be observed that both products show a unimodal peak. TiO_2_ possesses a wide pore size distribution according to BJH analysis, with most of the pores measuring around 8 nm, which is larger than the pore size of Co@TiO_2_ (7 nm). The pore volume of anatase TiO_2_ was determined to be 0.128 cm^3^ g^−1^. Compared with TiO_2_, the modified sample displays a larger pore volume of 0.238 cm^3^ g^−1^. These results indicate a significantly increased specific surface area and pore volume, which can provide more active sites for lithium-ion intercalation, improving the cycle stability and specific capacity of LIBs [40,41].

**Figure 3:**
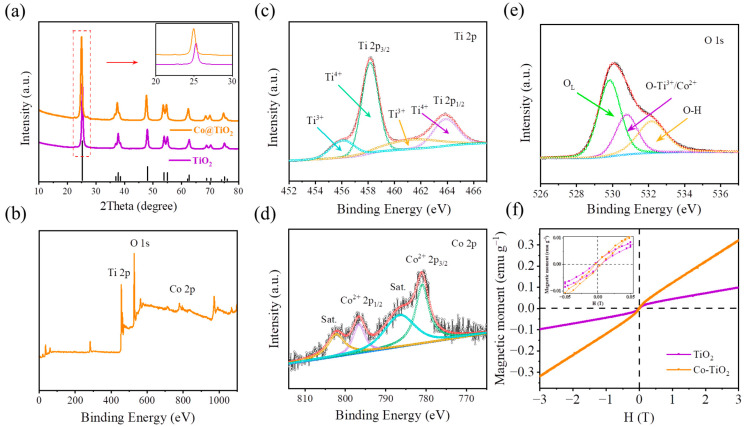
(**a**) XRD patterns of TiO_2_ and Co@TiO_2_. The inset in Figure 3a is the corresponding enlarged XRD pattern from the typical peak. (**b**) XPS spectra of Co@TiO_2_. XPS spectra of the Co@TiO_2_ sample: (**c**) Ti 2p, (**d**) Co 2p, and (**e**) O 1s. (**f**) Magnetic hysteresis loop for TiO_2_ and Co@TiO_2_ samples. The inset in Figure 3f shows the *M* (*H*) curve.

**Figure 4:**
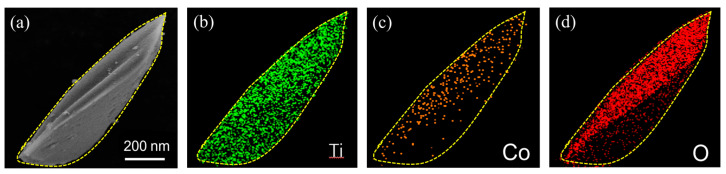
(**a**) SEM image of the Co@TiO_2_ and the corresponding elemental mapping for (**b**) Ti, (**c**) Co, (**d**) O. Elemental distributions in Co@TiO_2_.

The crystal structure of the samples was characterized by XRD. Considering that the crystal phase, grain size, and morphology of TiO_2_ are affected by temperature, anatase TiO_2_ with good crystallinity was synthesized using a wet chemical method [27]. Figure 3a illustrates the XRD patterns of TiO_2_ and Co@TiO_2_, demonstrating that the crystals are indexed to an anatase phase (JCPDS no. 21-1272). In contrast to TiO_2_, the inset of Figure 3a displays a slight shift in the (101) diffraction peak to the low angle for Co@TiO_2_, indicating that Co doping was achieved in TiO_2_ [39]. The surface chemical composition and element valence of Co@TiO_2_ were determined by XPS, as shown in Figure 3b. From the XPS survey spectrum of Co@TiO_2_, it is obvious that Co, Ti, and O are found on the surface of the composite. Figure 3c shows that the Ti spectrum of the Co@TiO_2_ sample can be fitted with four peaks corresponding to Ti^4+^ 2p_3/2_ at 458.1 eV, Ti^4+^ 2p_1/2_ at 463.82 eV, Ti^3+^ 2p_3/2_ at 456.05 eV, and Ti^3+^ 2p_1/2_ at 461.65 eV, the line separation between Ti^4+^ 2p_1/2_ and Ti^4+^ 2p_3/2_ being 5.72 eV, which is consistent with the standard binding energy of TiO_2_ [42]. However, the occurrence of Ti^3+^ peaks may be related to the Co doping content [43]. Figure 3d shows that the Co peak can be deconvoluted into two peaks: one at 780.8 eV, which corresponds to Co 2p_3/2_, and another at 796.2 eV, corresponding to Co 2p_1/2_ in Co 2p spectra, followed by two satellite peaks at approximately 786 eV and 802.5 eV, respectively. This further confirms the Co dopant in TiO_2_ [44]. The corresponding energy-dispersive X-ray spectroscopy (EDS) mapping also confirmed that TiO_2_ irregular prisms were uniformly doped by Co^2+^, as shown in Figure 4a–d. In addition, the content of Co^2+^ in the Co@TiO_2_ is about 10%. The deconvoluted O 1s spectrum (Figure 3e) is revealed by three Gaussian curves, which is consistent with the previous literature [45]. One accurate peak of O 1s, located at about 529.9 eV, arises due to the oxygen in the TiO_2_ crystal lattice (O_L_), while the other oxygen peaks, located at 530.9 eV and 532.1 eV, can be attributed to the outcome of Ti−O/Co−O bonds and the hydroxyl group (OH), respectively [43].

Magnetic characterization helps to understand the reaction mechanism of transition metals and provides valuable insights for the design of high-performance anodes [46,47,48,49,50]. In order to deeply understand the magnetic properties of TiO_2_ after Co doping, hysteresis loops were obtained by a Quantum Design PPMS magnetometer under ambient temperature, as shown in Figure 3f. Obviously, Co@TiO_2_ shows a greater magnetization, indicating the achievement of Co doping. The inset in Figure 3f shows that the variation is almost identical in the coercivity (*H*_C_) of the two samples, which confirms the ferromagnetic ordering at room temperature. However, it is known that pure TiO_2_ is diamagnetic. So far, there is no unified explanation for the cause of ferromagnetism in diamagnetic materials. There are two mainstream views; one explains this fact by the model of bound polarons [51,52], and the other attributes it to the existence of intrinsic point defects (oxygen vacancy, Ti vacancy, or Ti^3+^) [53,54,55,56,57,58,59]. Here, the ferromagnetism of TiO_2_ may be caused by oxygen vacancy/Ti^3+^ according to the analysis in Figure 3c,e. The above results indicate that Co may enter the lattice of TiO_2_, thereby improving the intrinsic electrical conductivity inside the TiO_2_ bulk. Meanwhile, more active materials may be activated due to the existence of Co, which helps to enhance lithium storage performance, especially in terms of capacity.

Over the past few decades, compared with rutile and TiO_2_ (B), anatase has been the most extensively studied because of its stable nanoscale crystal phase [5,60]. A variety of basic tests of the electrochemical performance of the products were conducted to evaluate their potential applicability in LIBs. The anatase phase of TiO_2_ can accommodate lithium ions via the electrochemical reaction. The insertion/extraction of Li^+^ ions in the crystal structure is performed according to the following equation [5,27]:

*TiO*_2_ + *x Li*^+^ + *x* e^−^ ↔ *Li_x_TiO*_2_

In terms of the anatase phase, the theoretical capacity of TiO_2_ will attain 336 mAh g^−1^ when *x* equals 1. However, the mole of electrons transferred is usually about 0.5 in electrochemical reaction [27,61].

### 3.2. Electrochemical Performance and Kinetic Analysis

**Figure 5:**
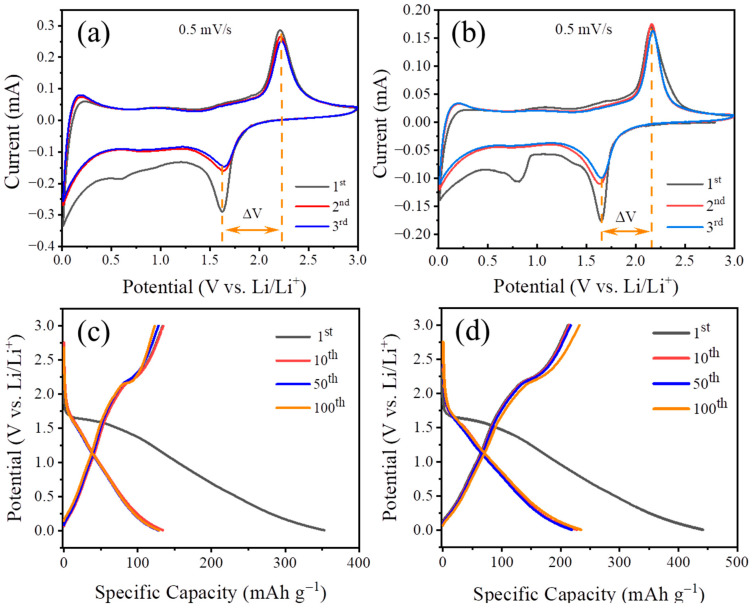
CV curves of the TiO_2_ (**a**) and Co@TiO_2_ (**b**) electrodes at a scan rate of 0.5 mV s^−1^. Discharge–charge potential profiles of the TiO_2_ (**c**) and Co@TiO_2_ (**d**) electrodes for the 1st, 10th, 50th, and 100th cycles over the potential window 0.01–3.0 V at 3 C.

The lithium-ion battery is composed of TiO_2_ or Co@TiO_2_ as the anode and lithium metal as the reference electrode. Figure 5a,b show the CV curves of TiO_2_ and Co@TiO_2_ electrodes at a scan rate of 0.5 mV s^−1^ for the first three cycles, respectively. The initial potentials of TiO_2_ and Co@TiO_2_ are 2.6 V and 2.7 V, respectively. It is obvious that, other than the first curve, the next four curves nearly overlap, demonstrating the highly reversible nature of the assembled battery. As shown in Figure 5a, a couple of redox peaks at ~1.62 V and ~2.22 V are observed from CV measurement, which can be attributed to the lithium ions intercalation into and deintercalation from the interstitial sites of TiO_2_. Figure 5b shows two reduction peaks located at 0.8 V and 1.65 V, respectively, and one oxidation peak located at 2.15 V in the first cycle. The reduction peak at 0.8 V should be attributed to the formation of a solid electrolyte interphase (SEI) film [39], which may be related to the catalysis of the Co [50]. Compared with TiO_2_, the separation between reduction and oxidation peaks (ΔV) of the modified sample decreases slightly, indicating that Co doping can reduce the polarization and impedance at the electrode and electrolyte interface, which is conducive to a better charge/discharge reversibility [27,39]. Moreover, the intensity of the oxidation peak decreases and shifts to a lower potential after Co doping. This phenomenon indicates the increase in lithium ion insertion dynamics and wettability of the Co@TiO_2_ electrode material, which is due to the decrease in the band gap under the action of Co [39]. Figure 5c,d shows the charge/discharge potential profiles of both TiO_2_ and Co@TiO_2_ electrodes for the 1st, 5th, 10th, and 100th cycles at 3 C between 0.01 and 3 V. Obviously, Co@TiO_2_ exhibits a higher discharge specific capacity of 441 mAh g^−1^ in the initial discharge process, the corresponding charge capacity being 227 mAh g^−1^ (Figure 5c). As for TiO_2_, it shows a lower capacity of 353 mAh g^−1^ with a corresponding charge capacity of 133 mAh g^−1^, as shown in Figure 5d. Obviously, for Co-doped TiO_2_, the doping of cobalt metal can provide capacity, which may come from the redox reaction [62,63] and interface space charge [51,64]. This theoretical capacity is closely related to the content of cobalt and is difficult to quantify. The first coulombic efficiencies (CE) of 51.5% and 37.7% were obtained for Co@TiO_2_ and TiO_2_, respectively. Noting that the initial CE of Co@TiO_2_ is higher than that of TiO_2_, which is mainly due to the improvement in the electronic conductivity of TiO_2_ after the transition metal doping [39,65]. Notably, both exhibit an irreversible capacity loss during the first cycle, which can be attributed to the formation of the SEI film [66,67]. Fortunately, the irreversible capacity of Co@TiO_2_ gradually decreases in the subsequent cycle, indicating that the electrochemical reaction becomes stable in the process of lithium insertion/extraction. This can be ascribed to the enhancement in structural stability and electronic conductivity by Co doping. In addition, TiO_2_ shows larger irreversible capacity decay, which may be caused by its inherent poor conductivity.

**Figure 6:**
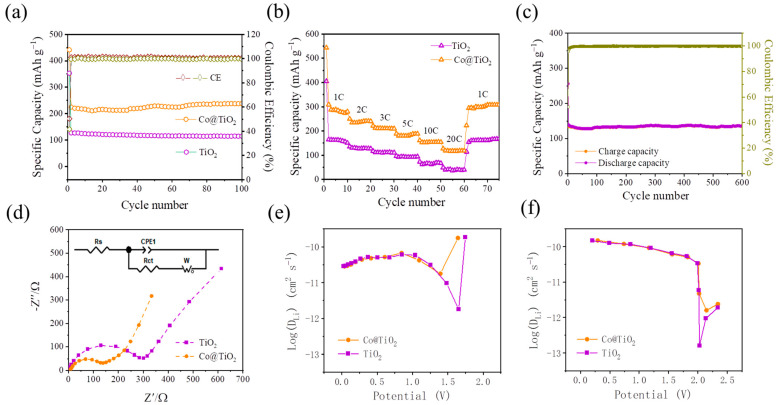
(**a**) Cycling performance over the potential window 0.01–3.0 V at 3 C. (**b**) Rate performance at various current rates of TiO_2_ and Co@TiO_2_ over the potential window 0.01–3.0 V. (**c**) Cycling performance of the Co@TiO_2_ sample over the potential window 0.01–3.0 V at 10 C. (**d**) EIS of the Co@TiO_2_ and TiO_2_ electrodes. (**e**,**f**) GITT curves and *D*_Li_ values of the Co@TiO_2_ and TiO_2_ electrodes.

The electrochemical properties of TiO_2_ and Co@TiO_2_ were evaluated in assembled LIBs with lithium metal counter electrodes and CR2032 coin battery geometry. The cycling capacity of both TiO_2_ and Co@TiO_2_ at a current density of 3 C between 0.01 V and 3 V is shown in Figure 6a. The capacity of TiO_2_ decreases gradually. Compared with TiO_2_, Co@TiO_2_ has better cycle performance. Moreover, it is clearly observed that the cyclic retention of Co@TiO_2_ with a reversible capacity of 237 mAh g^−1^ is significantly higher than that of TiO_2_ (114 mAh g^−1^) after 100 cycles. Due to the difference between the oxidation states of cobalt (Co^2+^) and titanium (Ti^4+^), Co doping introduces lattice distortion and oxygen vacancies in the crystal structure, which helps to reduce the band gap and improve the electron transport rate [68]. Even after 600 cycles, the Co@TiO_2_ electrode still provides a stable and high reversible capacity of 125 mAh g^−1^ at a heavy current rate of 10 C, which is at a relatively high level compared with those of the reported anode materials, as shown in Figure 6c. The details are described in Table 1. Figure 6b exhibits an excellent rate performance of Co@TiO_2_ at charge–discharge rates between 1 C and 20 C. The specific capacities of 274, 240, 209, 180, and 147, 117 mAh g^−1^ can be delivered at current densities of 1, 2, 3, 5, 10, and 20 C, respectively. When the current density returns to 1 C, a high specific capacity of 275 mAh g^−1^ can be maintained. These results clearly demonstrate that the Co@TiO_2_ has excellent lithium storage performance with long cycle life and great rate capability for the fast charge–discharge process. In short, the excellent lithium storage property of Co@TiO_2_ as an anode material for LIBs can be attributed to several aspects. First of all, a higher BET specific surface area, and more electrochemical active sites and electrolyte/electrode interfaces, promote better electrolyte permeation and provide more possibilities for lithium storage. Secondly, the porous structure of electrode materials has the advantage of not only shortening the distance of ion transport, but also buffering severe volume fluctuation during lithiation/de-lithiation. Thirdly, the smaller impedance and larger diffusion coefficient can accelerate the electrochemical kinetics. Additionally, the increased electrical conductivity can promote electron transport. All these features demonstrate that Co@TiO_2_ has an enhanced electrochemical performance, especially in terms of capacity.

In order to investigate the kinetic properties of the TiO_2_ and Co@TiO_2_, EIS and GITT were conducted. As presented in Figure 6d, both Co@TiO_2_ and TiO_2_ have similar characteristics, including electrolyte resistance (R_s_) at high frequencies, a depressed semicircle at the middle frequencies corresponding to the charge transfer process (R_ct_), and a straight line at low frequencies caused by Warburg impedance (W) reflecting the solid-state diffusion of lithium ions in the electrode. Both curves are fitted with a typical equivalent circuit (the inset of Figure 6d). According to the fitting impedance data, R_ct_ of 300 Ω and 140 Ω was obtained for TiO_2_ and Co@TiO_2_, respectively. Obviously, the charge transfer resistance of the Co@TiO_2_ electrode is much lower, almost half that of the TiO_2_ electrode. This is due to the increase in conductivity, which should come from the mixed valence state induced by the Co^2+^ ions [69].

Furthermore, the GITT was performed to research the lithium diffusion coefficient (*D*_Li_), as shown in Figure 6e. Obviously, the change in *D*_Li_ strongly depends on the charge and discharge process. The *D*_Li_ value of TiO_2_ in the initial discharge is 1.88 × 10^−10^ cm^2^ s^−1^. Subsequently, *D*_Li_ decreases rapidly and achieves a minimum value of 1.76 × 10^−12^ cm^2^ s^−1^. Next, *D*_Li_ increases to 6.05 × 10^−11^ cm^2^ s^−1^. In the end, *D*_Li_ slightly decreases again to 2.90 × 10^−11^ cm^2^ s^−1^. As can be seen from Figure 6e, Co doping significantly improves the *D*_Li_ of TiO_2_. In particular, the minimum *D*_Li_ is 1.79 × 10^−11^ cm^2^ s^−1^ in the reduction platform region, about 10 times higher than that of TiO_2_. Similarly, Co@TiO_2_ shows a faster ion diffusion rate in the oxidation plateau region than TiO_2_ (Figure 6f). The results show that Co doping enhances the dynamic performance of TiO_2_, thereby improving the overall electrochemical performance [36]. 

## 4. Conclusions

In conclusion, Co-doped anatase TiO_2_ has been successfully synthesized with superior reversible capacity by a hydrothermal method. It is worth noting that the Co^2+^-doped TiO_2_ not only possesses a large specific surface area, pore volume, and mesoporous structure with narrow pore distribution, but also has enhanced conductivity. It is found that doping Co^2+^ in the TiO_2_ anode can improve its reversible capacity, rate performance, and coulombic efficiency. Compared with undoped TiO_2_, the Co@TiO_2_ electrode shows superior electrochemical performance. Therefore, as an advanced anode material for lithium storage, it exhibits high capacity and good cycling ability. The performance of electrode materials can be greatly improved by doping, which can promote the commercial application of TiO_2_ anode materials.

## Figures and Tables

**Table 1:** The comparison of different doped TiO_2_ as anode for LIBs.

Anode	Cycle Performance	Rfs (Year)
Fe–S-doped anatase TiO_2_ nanotubes	61.4 mAh g^−1^ at 1680 mA g^−1^ after 500 cycles	[20] (2016)
Fe-doped anatase TiO_2_/carbon composite Europium-modified TiO_2_ Mn-doped TiO_2_ Cu-doped TiO_2_ B-doped TiO_2_ F-doped carbon coated mesoporous TiO_2_ Co-doped TiO_2_	158.6 mAh g^−1^ at 1700 mA g^−1^ after 300 cycles 219.1 mAh g^−1^ at 850 mA g^−1^ after 600 cycles 113 mAh g^−1^ at 170 mA g^−1^ after 118 cycles 250 mAh g^−1^ at 500 mA g^−1^ after 100 cycles 119.4 mAh g^−1^ at 1680 mA g^−1^ after 100 cycles 210 mAh g^−1^ at 84 mA g^−1^ after 100 cycles 125 mAh g^−1^ at 1700 mA g^−1^ after 600 cycles	[27] (2015) [28] (2022) [29] (2020) [30] (2018) [31] (2014) [33] (2014) In this work

## Data Availability

Not applicable.
